# A New Take on V(D)J Recombination: Transcription Driven Nuclear and Chromatin Reorganization in Rag-Mediated Cleavage

**DOI:** 10.3389/fimmu.2013.00423

**Published:** 2013-12-06

**Authors:** Julie Chaumeil, Jane A. Skok

**Affiliations:** ^1^Department of Pathology, New York University School of Medicine, New York, NY, USA

**Keywords:** V(D)J recombination, transcription, nuclear organization, higher-order loops, ATM, nucleosomes, RAG, pericentromeric heterochromatin

## Abstract

It is nearly 30 years since the Alt lab first put forward the accessibility model, which proposes that cleavage of the various antigen receptor loci is controlled by lineage and stage specific factors that regulate RAG access. Numerous labs have since demonstrated that locus opening is regulated at multiple levels that include sterile transcription, changes in chromatin packaging, and alterations in locus conformation. Here we focus on the interplay between transcription and RAG binding in facilitating targeted cleavage. We discuss the results of recent studies that implicate transcription in regulating nuclear organization and altering the composition of resident nucleosomes to promote regional access to the recombinase machinery. Additionally we include new data that provide insight into the role of the RAG proteins in defining nuclear organization in recombining T cells.

## Introduction

V(D)J recombination occurs during lymphocyte development to create B and T cell receptors that can recognize a vast array of foreign antigen. Diversity is generated within the seven antigen-receptor loci (four T cell receptor loci, *Tcrg, Tcrd, Tcrb*, and *Tcra* and three immunoglobulin loci, *Igh, Igk*, and *Igl*) by reshuffling variable (V), diversity (D), and joining (J) gene segments that are arrayed along the length of each of these large loci. Rearrangement is mediated by the RAG recombinase, which binds to highly conserved heptamer and nonamer recombination signal sequences (RSSs) that flank each of the V, D, and J gene segments. Both RAG1 and RAG2 proteins, which make up the recombinase, bind to two segments, bringing them together to form a synapse prior to the introduction of double strand breaks (DSBs). In addition, RAG plays a role after cleavage by holding the four broken ends together in a RAG post cleavage complex that directs repair by the ubiquitous classical non-homologous end joining (C-NHEJ) pathway.

Although the process of rearrangement is common to all antigen-receptor loci and mediated by the same machinery, it is regulated so that *Ig* and *Tcr* loci are respectively rearranged at the appropriate stage of B and T cell development. Furthermore, cleavage is restricted at the allelic level (allelic exclusion) to ensure rearrangement and cell surface expression of a single specificity receptor. Studies from numerous labs have validated the accessibility model put forward by the Alt lab and shown that rearrangement is linked with transcription, active histone modifications, and reversible locus contraction, which brings widely separated gene segments together by looping (1, 2).

## RAG Binding and Distribution within the Nucleus

Both RAG1 and RAG2 are required for cleavage although the endolytic activity lies within the RAG1 protein. RAG2 binds to chromatin via its PHD domain, which specifically recognizes the histone modification, H3K4me3 (3, 4). Genome wide ChIP-seq analyses indicate that RAG2 recruitment mirrors the footprint of this active histone modification. In contrast, RAG1 binding is more directed and occurs predominantly at conserved RSSs (5), however binding can also occur at cryptic RSS sites that are scattered throughout the genome. As RAG binding is not limited to the antigen-receptor loci alone, this raises a question about the mechanisms that direct cleavage. Clearly, DSBs are not introduced everywhere in the genome at sites of active chromatin or indeed at consensus/cryptic RSSs, so there must be other factors involved in determining when breaks are generated.

One possibility to consider, beyond active chromatin and the nature of the RSS, is the localized concentration of RAG1/2. It is logical to assume that the higher the concentration of recombinase in the vicinity of a vulnerable gene, the more likely the chances of cleavage. RAG2 localizes to euchromatic regions of the nucleus and domains of RAG enrichment are clearly visible by microscopy after immunostaining (Hewitt and Skok, unpublished). But what is the mechanism underlying the generation of these focal centers? The data from recent genome wide chromosome conformation capture experiments indicates that co-regulated actively transcribed genes come together in the nucleus in transcription factories (6, 7). Thus, contact between common transcription factor or RAG bound loci will likely increase the local concentration of these factors in the nucleus, as shown for polycomb bound regions that associate to form a polycomb body (8). Since gene expression depends on the integrated binding of a number of different remodeling and transcription factors, the balance of these will likely determine which factors are dominant in defining the intra- and inter-chromosomal interaction partners of any particular locus.

## Population Versus Single Cell Analysis

**Figure 1:**
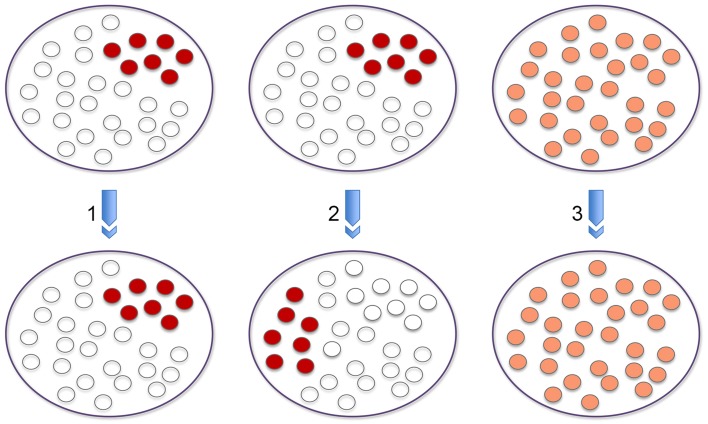
**Population versus single cell analysis**. The three panels provide different outcomes at the single cell level that explain signal enrichment from a population based chromosome conformation capture experiment that reflects interaction between two loci. Signal enrichment could reflect interaction at high frequency in only a subset of cells within the population (1). At a different time point this interaction could be occurring in the same or a different subset of cells within the population (2). A third possibility could be that interaction occurs at a roughly uniform frequency in every cell of the population (3) and this leads to an equivalent signal enrichment as the interactions in (1) or (2).

When considering the data from genome wide association studies it is important to remember that signal enrichment reflects the sum of the data derived from a population of cells. What happens at the single cell level may be very different. However, without live systems in which we can track the movements of individual loci in single cells over a period of time, at the simplest level, when focusing on an interaction between two loci in a population, we cannot tell whether chromosome conformation capture signal enrichment reflects interaction at high frequency in only a subset of cells within the population (1); whether at a different time point this interaction will be occurring in the same (1) or a different subset of cells within the population (2); or whether interaction occurs at a roughly uniform frequency in every cell of the population (3) and this leads to an equivalent signal enrichment as the interactions in (1) or (2) (Figure 1). Nonetheless, in the absence of these live systems we can address some of these questions using single cell DNA FISH analyses on a population of fixed cells. With this approach, although we cannot distinguish between (1) and (2), we can distinguish between these two alternatives and (3) by determining whether interactions occur at a similar frequency in every cell versus a high frequency in a subset of cells. The same issues arise for histone modifications and transcription factor/RAG binding at a particular site. Furthermore, if genome wide data sets are integrated, e.g., chromosome conformation capture and ChIP-seq, the situation becomes even more complex.

## The Role of RAG in Inter-Chromosomal Interactions

To examine these issues in the context of V(D)J recombination, we asked whether RAG binding could have a role in bringing RAG bound antigen-receptor loci together in the nucleus in localized recombination centers. We discovered that expression of RAG1 brings target homologous antigen-receptor alleles together in a subset of recombining cells (9–11). Homologous pairing of *Ig* or *Tcr* alleles occurs prior to and independent of RAG cleavage because expression of a catalytically inactive RAG1D708A mutant protein can rescue pairing in RAG1-deficient cells. In addition to increasing the local concentration of RAG in the nucleus, a second not mutually excusive possibility is that communication between the two alleles could be important for regulation of cleavage on homologs. Indeed, we found that the introduction of a break on one allele halts the introduction of further breaks on the second allele through the action of the DNA damage response factor Ataxia telangiectasia mutated (ATM) (10, 11). Briefly, ATM, recruited to the site of a break on one allele, acts *in trans* on the second allele repositioning it to pericentromeric heterochromatin (PCH). Transient relocation to this repressive nuclear environment likely causes a degree of silencing that depletes RAG binding on the uncleaved allele during repair of the first break. Thus, ATM-mediated changes in nuclear organization function to ensure asynchronous RAG-mediated cleavage on homologous alleles. Regulation *in trans* is important for the initiation of allelic exclusion and for restricting the number of DSBs that are introduced at any one time in the cell (10). Based on our results we favor a model in which RAG-mediated breaks are introduced on closely associated homologs and then separate for repair to facilitate regulated asynchronous cleavage. However, without a live imaging system in which we can track the dynamics of cleavage and repair, we cannot definitively determine whether this is the case. Nevertheless, it is clear that if homologs are paired the uncleaved allele will have immediate access to a high concentration of activated ATM recruited to the site of damage on the cleaved allele.

## Higher-Order Loop Formation during Recombination

**Figure 2:**
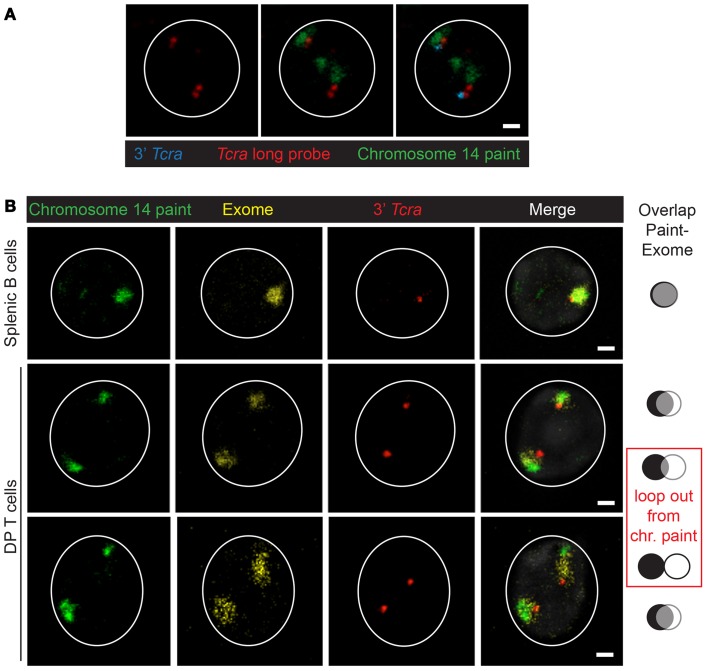
**Organization of the chromosome 14**. **(A)** Example of looping out of *Tcra/d* from the chromosome 14 territory in wild-type DP cells. Chromosome 14 paint in green, long *Tcra/d* probe in red, 3′ *Tcra* probe (BAC RP23-255N13) in blue. Scale bar = 1 μm. **(B)** Examples showing the 3D-organization of the chromosome 14 territory with a chromosome paint (repetitive sequences) in green, an exome probe (gene-rich regions) in yellow and the 3′ *Tcra* probe in red. The paint and exome domains overlap in splenic B cells and in DP T cells when *Tcra* is close to the paint domain and not looped out, while the two signals are more separated when *Tcra* is looped out from the paint. In either case *Tcra* is never looped out from the exome domain. Scale bar = 1 μm.

Beyond locus contraction and homolog pairing we recently uncovered an additional layer of regulation involving nuclear organization that occurs during V(D)J recombination: the formation of higher-order loops (11). Chromosomes occupy discreet territories in interphase cells and the size and position of these within the nucleus is dependent on the cell type and developmental stage (12). Live imaging studies have shown that chromosome territories move very little following mitosis (13), so gene mobility facilitated by the formation of higher-order loops provides an opportunity for loci on different chromosomes to contact each other in nuclear space. Movement of genes away from their individual chromosome territories linked to activation/transcription (14) has been shown to facilitate stochastic inter-chromosomal interactions (15), but little is known about whether pairing of this sort could be involved in regulation of genes *in trans*. Our data indicate that, as with homolog pairing, RAG1 expression (independent of its catalytic activity) induces the formation of higher-order loops that separate the 3′ end of the antigen-receptor locus, *Tcra*, from its 5′ end which remains embedded in the chromosome territory (as assessed by DNA FISH with a chromosome paint probe) (11) (Figure 2A). Furthermore, *Tcra* expression is linked to looping and pairing because in splenic B cells where RAG is not present and *Tcra* is not transcribed loop formation is inhibited and the two loci pair at a frequency below the levels seen in RAG1-deficient cells. Additional RNA/DNA FISH analyses revealed that the proportion of looped out alleles that are transcribed is greater than those located at the outer edge of the chromosome territory, while those alleles that are buried in the territory are not associated with any RNA signal at all. It should be noted that although we used an oligonucleotide probe pool covering the entire locus except for the most repetitive regions, nascent RNA signals could only be detected at the 3′ end of *Tcra*, likely because with this assay there is a threshold below which transcription cannot be detected. Furthermore, we found that RNA signals are not distributed uniformly throughout the population: 1/3 of the cells had no *Tcra* signals, 1/3 of the cells had one *Tcra* signal and the remaining third had signals from both alleles. Thus, *Tcr* transcription of 3′ *Tcra* occurs in a subset of cells and this is linked with looping out of this region.

To examine in further detail how the chromosome territory and the locus are organized in nuclear space, we designed an oligonucleotide probe pool covering only the exon sequences represented on chromosome 14 (called “exome”) (16). We used the exome in conjunction with a conventional chromosome 14 paint (that largely encompasses repetitive sequences), as well as the 3′ *Tcra* BAC probe. Curiously, we found considerable overlap between the paint and exome DNA probes for chromosome 14 in splenic B cells and in DP T cells on chromosomes where *Tcra* is not looped out (Figure 2B). In contrast there was very little overlap between the paint and exome in DP cells on the allele on which *Tcra* forms higher-order loops (Figure 2B). These observations indicate that looping out of *Tcra* extends exome sequences, dragging them to the outside of the territory so that they no longer overlap with the paint signal. These data, underline the link between loop formation and transcription that we and others previously documented (11, 14, 17). The Bickmore lab previously showed that chromosome paints only reveal the core of chromosome territories while exome probes can detect gene-rich regions that are mostly located around the outside of these core domains (16). Here our analysis of chromosome 14 in two different cell types provides additional insights into the dynamics of chromosome organization, indicating that high levels of expression from an individual locus and the presence of a *trans* acting factor such as RAG, can impact on looping, with the gene-rich regions being reorganized outside of the core domain alongside the highly transcribed *Tcra*.

What is the purpose of higher-order loop formation in DP T cells? Interestingly, we found that looping out of *Tcra* occurs on only one allele and this was linked to the occurrence of RAG-mediated mono-allelic breaks on the 3′ end of the looped out of *Tcra* (11). These data link up with previous genome wide analyses from the Schatz lab showing that active histone marks and RAG1/2 binding are enriched at the 3′ region of recombining antigen-receptor loci (5). However, these are population derived data so they do not distinguish between signals in individual cells and on homologous alleles, thus it is not possible to determine whether RAG and active histone marks are enriched on antigen-receptor loci in a subset of cells and specifically whether RAG binding is more concentrated on the looped out allele. Nonetheless, it is clear that RAG binding coupled with a high level of transcription is linked to movement of the 3′ end of one allele away from the territory and this in turn correlates with the introduction of RAG-mediated mono-allelic cleavage on the looped out allele.

## Regulation of Mono-Locus Cleavage

**Figure 3:**
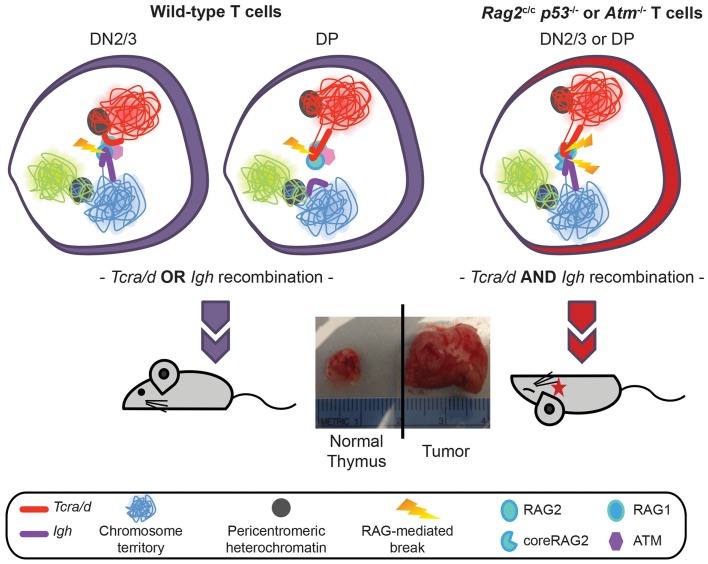
**Model showing regulation of *Tcra/d* and *Igh* mono-locus recombination by ATM and RAG2 C-terminus through modulation of nuclear organization**. In wild-type DN2/3 cells expression of RAG mediates pairing of *Igh* and *Tcra/d* at a high frequency. Association of the two loci occurs through the formation of RAG dependent higher-order loops on one locus. This organization is involved in mediating *trans* regulation and restricted cleavage: targeted RAG breaks are introduced at the 3′ end of the looped out locus while further cleavage events on the second locus are inhibited during repair of the first break. Regulated asynchronous recombination on the two loci in the same cell involves the C-terminus of the RAG2 protein and ATM (that is recruited to the site of a DSB RAG break). Both factors control cleavage on the second locus by repositioning the uncleaved locus to repressive pericentromeric heterochromatin, inhibiting the formation of higher-order loops, and decreasing the frequency of pairing. In the absence of the C-terminus of RAG2 or ATM the two loci remain euchromatic, loops can form on both, and they stay paired at high frequency. This results in the introduction of bi-locus breaks and damage on closely associated loci, which provides a direct mechanism for the generation of these inter-locus translocations that are a hallmark of ATM deficient and *Rag2*^c/c^ mice.

To extend these studies we asked whether similar mechanisms could control RAG cleavage on different loci undergoing recombination at the same stage in development. For this we analyzed the *Tcra/d* locus in conjunction with the *Igh* locus. Although *Tcrd* and *Tcra* occupy the same chromosomal location (*Tcrd* is embedded within the *Tcra* locus) the two loci undergo recombination at different stages of T cell development in CD4 CD8 double negative, DN2/3, and CD4 CD8 double positive DP cells, respectively. For reasons that are not well understood, the *Igh* locus undergoes a low level of partial rearrangement in T lineage cells and in this context it is of note that *Igh* has been identified as a translocation partner of *Tcra/d* in T lineage lymphomas. Specifically, we and others have found that *Tcra/d-Igh* translocations occur in T-lymphomas from ATM deficient mice (18–21). Furthermore, we recently discovered T-lymphomas with *Tcra/d*-*Igh* translocations in mice expressing a truncated version of RAG2, missing the non-core regulatory C-terminal domain crossed onto a p53 deficient background (18). Our recent investigations have revealed that ATM and the RAG2 C-terminus prevent bi-locus RAG-mediated cleavage through similar mechanisms: modulation of three-dimensional conformation (higher-order loops) and nuclear organization of the two loci (22). Thus, the RAG2 C-terminus and ATM control asynchronous RAG cleavage on homologous and heterologous antigen-receptor alleles in a similar manner: through repositioning the uncleaved allele/locus at repressive PCH, inhibiting bi-allelic/bi-locus loop formation and bi-allelic/bi-locus cleavage. This limits the number of potential substrates for translocation and provides an important mechanism for protecting genome stability (Figure 3) (11, 22).

## RAG Brings Recombining Loci Together in the Nucleus

Interestingly, control of cleavage of *Tcra/d* and *Igh* in T cells involves RAG-mediated regulation of association of the two loci. Thus, it appears that RAG brings recombining loci together in the nucleus. In this case the two loci are close together in DN cells (when both *Tcrd* and *Igh* are recombining) but interact far less frequently in DP cells (when *Igh* is recombined at lower levels). This raises the possibility that feedback control of RAG activity could involve close communication between heterologous antigen-receptor alleles as we have proposed for homologous alleles. Either way, it appears that regulation *in trans* involves control of loop formation and repositioning of the other allele/locus to a repressive compartment of the nucleus.

**Figure 4:**
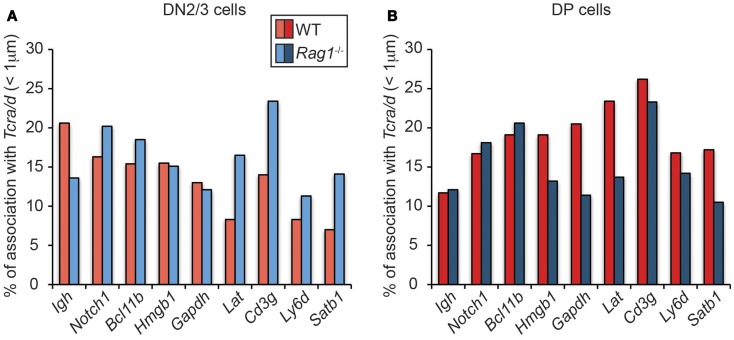
**Dynamics of *Tcra* association with H3K4me3- enriched active loci in WT and RAG1-deficient DN2/3 and DP cells**. DNA FISH was performed using fluorescent BAC probes for *Tcra, Igh*, and the H3K4me3-enriched active loci, images were recorded on a Leica SP5 confocal and distances were measured in 3-dimension using Image J software. Association was defined as a distance between the two centers of mass under 1 μm in DN2/3 **(A)** and DP **(B)** cells.

Given these findings and the fact that RAG2 can bind to H3K4me3-enriched active loci throughout the genome, we considered the possibility that there could be localized feedback control through association of RAG enriched loci. However, when we looked to see whether RAG induces association of *Tcra/d* with other hematopoietic lineage specific or housekeeping H3K4me3-enriched loci on different chromosomes in DN2/3 cells (where *Tcrd* and *Igh* are recombining), we found an opposite trend to the relationship between *Igh* and *Tcra/*d (Figure 4A), and as shown previously (22): depletion of RAG1 in the cells increased rather than decreased association of these other genes with *Tcra/d*. This trend was mirrored by *Notch1* and *Bcl11b* that we newly examined here: if anything, RAG1 expression separates these loci from recombining *Tcrd* at the DN2/3 stage of development. This is of interest because RAG targeting of both these loci is linked to lymphoid malignancies (23). In contrast, when we examined interactions in DP cells (where *Tcra* is recombining) while RAG depletion did not alter the relationship between *Igh* and *Tcra/d* (or *Notch1* and *Bcl11b*) in many instances it increased the association of the other genes with *Tcra/d* (Figure 4B). Moreover, in DP cells, *Tcra/d* was always closer to the other loci analyzed compared to *Igh*. It is interesting to note that even though *Bcl11b* is located adjacent to *Igh* on chromosome 12, the two loci associate with *Tcra/d* at different frequencies. In sum, these data indicate that RAG differentially influences the spatial relationship of RAG enriched loci at the DN and DP stages of development. The change in trend in the two cell types could be influenced by the enrichment of bound RAG, the level of transcription, and differences in the transcription factor binding profiles of the individual genes in these cells. What is clear though is that there is a shift in organization of these loci at the two different stage of development that in many cases is affected by the presence of RAG1.

## Transcription Mediated Nucleosome Reconfiguration Provides Transient RSS Access to RAG

Our data linking transcription with altered nuclear organization and targeted RAG cleavage of *Tcra* (11), go hand in hand with another recent paper that demonstrates the significance of transcription and altered chromatin organization in targeting of RAG-mediated breaks (24). Bevington and Boyes examined the requirements for activation of the *Igk* and *Igl* light chain loci by making use of interferon regulatory factor IRF4/IRF8 double deficient mice, which are blocked at the pro- to pre-B cell stage of development (25). One of the major defects of an absence of IRF4 is the loss of activation of enhancer elements on the *Igk* and *Igl* light chain loci, which are important for rearrangement (26). Restoration of light chain rearrangement and non-coding transcription can occur by enforced transgenic overexpression of IRF4 in *Irf4*^−/−^ *Irf8*^−/−^ double mutant mice at the earlier pro-B cell stage of development. However, in contrast to wild-type pre-B cells (where the *Igk* locus is recombined before *Igl*), the *Igl* locus is rearranged in preference to *Igk* in the transgenic mice. This *in vivo* model enabled them to examine the mechanisms underlying RSS accessibility at these two loci. Surprisingly, they find that high levels of H3K4me3 at Jκ RSSs only leads to partial activation of recombination at the *Igk* locus indicating that enrichment of this mark is not tightly linked to recombination. Furthermore, they show that neither H3 and H4 histone acetylation is sufficient to increase RSS accessibility although enrichment of histone acetylation appears to be linked to early activation of the two loci. In addition they find no correlation between H3K36me3 enrichment and recombination at the *Igl* locus. Thus, none of the chromatin modifications that are associated with recombination are tightly linked to increased RSS accessibility. In contrast, increased accessibility, as measured by restriction enzyme digestion close to RSS sites in *Igl* versus *Igk*, correlates well with increased *Igl* recombination. Importantly, inhibition of transcription, through treatment with α-amanitin, abrogates restriction enzyme accessibility.

**Figure 5:**
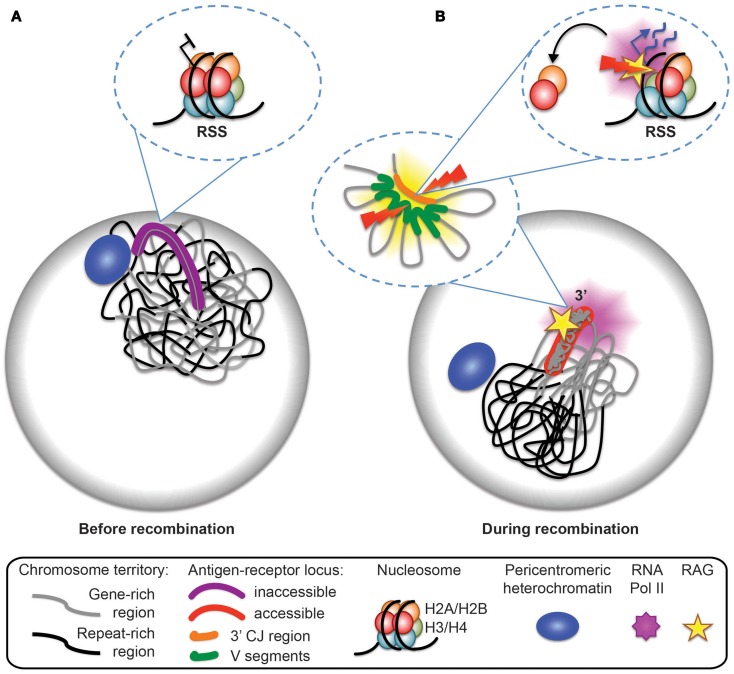
**Model for antigen-receptor locus accessibility to the RAG complex at the time of recombination**. **(A)** In non-recombining cells, the antigen-receptor locus is inactive, decontracted, and stays within its chromosome territory. It is located at the nuclear periphery or close to pericentromeric heterochromatin (PCH). **(B)** At the time of recombination, the antigen-receptor locus leaves the vicinity of the nuclear periphery or PCH, becomes contracted (forming internal loops), loops out from the repeat-rich core of the chromosome territory, and becomes accessible to the RAG machinery. Transcription dependent eviction of H2A/H2B dimers from the nucleosomes and the formation of hexamers provides a mechanism for RAG access at recombination signal sequences (RSS).

To further explore the links between recombination, restriction enzyme digest efficiency and transcription Bevington and Boyes analyze changes in nucleosome composition as a potential mechanism for increasing RSS accessibility. Specifically they examine eviction of an H2A/H2B dimer from nucleosomes, which temporarily converts the latter to hexasomes during the passage of RNA polymerase (27–29). Nucleosome to hexasome conversion is known to reduce histone/DNA contacts and release 35–40 bp of DNA, which could permit RAG access to RSS sites. To test this prediction they analyzed uncoupled cleavage on *in vitro* substrates and show that RAG is more efficient in cleaving hexasomes than nucleosomes. Furthermore, they demonstrate that there is an inverse relationship between elevated levels of RNA polymerase II and the presence of H2A/H2B (see model in Figure 5). RSS accessibility is transient, which fits with the observed displacement time for H2A/H2B eviction of 6 min during passage of RNA polymerase II (27). In the context of recombination, transient RSS accessibility implies that only a limited number of RSSs will be available for recombination at any one time.

Bevington and Boyes speculate that the transient nature of RSS accessibility may be important to inhibit excess RAG cutting and genome instability. This fits well with our data showing that mono-allelic RAG cleavage occurs on the 3′ end of looped out *Tcra* alleles, and that looping out occurs in only a subset of cells and thus it is likely to be a transient event (see model in Figure 5). Furthermore, we demonstrate that looping out is linked to high levels of transcription at the 3′ end of *Tcra* and is dependent on the presence of RAG1. Feedback regulation of RAG cleavage by ATM and the C-terminus of RAG2 involve changes in nuclear accessibility that include inhibition of higher-order loop formation and repositioning of the uncleaved antigen-receptor allele to repressive PCH. In the absence of these changes we find an increase in bi-allelic and bi-locus cleavage and genome instability.

## Concluding Remarks

With the current systems that are available it is difficult to study the cascade of interdependent events that occur during V(D)J recombination, particularly since many of these maybe very transient. The challenge now is to develop live cell systems in which cleavage and repair can be analyzed over a period of time in single cells. This is no trivial matter when it comes to the antigen-receptor loci as these are spread over megabases of DNA. In addition to labeling of different regions along the loci, the RAG1/2 recombinase and components of the DNA damage response and repair machineries need to be tagged for visualization. Until such systems are in place the next best approach is to focus on single cell analyses.

## Conflict of Interest Statement

The authors declare that the research was conducted in the absence of any commercial or financial relationships that could be construed as a potential conflict of interest.
